# Fluoxetine partially alleviates inflammation in the kidney of socially stressed male C57 BL/6 mice

**DOI:** 10.1002/2211-5463.13670

**Published:** 2023-08-10

**Authors:** Hailong Jin, Guanglei Xu, Yuchen Lu, Chunxiao Niu, Xueting Zhang, Tongtong Kan, Junxia Cao, Xiqin Yang, Qianqian Cheng, Jiyan Zhang, Jie Dong

**Affiliations:** ^1^ The Third Center PLA General Hospital Beijing China; ^2^ Beijing Institute of Basic Medical Sciences China

**Keywords:** depression, fluoxetine, inflammation, kidney, transcriptome

## Abstract

Stress‐related illnesses are linked to the onset and progression of renal diseases and depressive disorders. To investigate stress‐induced changes in the renal transcriptome associated with the development of depressive behaviors, we generated here a chronic social defeat stress (CSDS) model of C57 BL/6 male mice and then performed RNA sequencing of the kidneys to obtain an inflammation‐related transcriptome. Administration of the antidepressant drug fluoxetine (10 mg·kg^−1^·day^−1^) during CSDS induction could partially alleviate renal inflammation and reverse CSDS‐induced depression‐like behaviors. Moreover, fluoxetine also modulated gene expression of stress‐related hormone receptors, including prolactin and melanin‐concentrating hormone. These results suggest that CSDS can induce gene expression changes associated with inflammation in the kidney of C57 BL/6 male mice, and this inflammation can be treated effectively by fluoxetine.

## Graphical abstract

Psychological stress is involved in renal diseases and depressive disorders. We performed RNA sequencing of a chronic social defeat stress (CSDS) model of C57 BL/6 male mice to obtain a renal inflammation‐related transcriptome and report that renal inflammation was partially alleviated by the antidepressant drug fluoxetine. Moreover, fluoxetine modulated gene expression of stress‐related hormone receptors, including prolactin and melanin‐concentrating hormone.

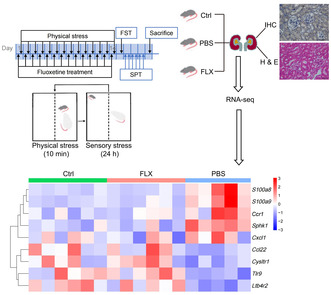

**Abbreviations**
BP: biological process
CKD: chronic kidney disease
CSDS: chronic social defeat stress
Ctrl: control
FLX: fluoxetine
FPKM: fragments per kilobase per million mapped reads
FST: forced swim test
GO: Gene Ontology
GSEA: gene set enrichment analysis
H & E: hematoxylin and eosin
IHC: immunohistochemistry
PBS: phosphate‐buffered saline

In 2022, the report launched by the World Health Organization (WHO) showed that mental disorders such as depression and anxiety had increased by more than 25% due to the COVID‐19 pandemic, adding to the nearly one billion people with a mental disorder [1]. Depression is an already‐common and recurrent mental disorder, recognized as one of the leading causes of disability and global disease burden [2]. Depression is a multi‐factorial mental disturbance, which is partially attributed to excessive or prolonged psychological stress [3, 4, 5]. Up to now, the exact mechanisms underlying the pathogenesis of depression remain to be unraveled. However, it has been reported that psychological stress induces a pro‐inflammatory response, which might affect peripheral tissues and organs, as well as the brain, and subsequently, inflammation contributes to several psychiatric diseases, such as depression [6, 7]. Neurotransmitter serotonin is synthetized from its sole precursor tryptophan and influences a variety of behavioral functions [4, 5, 8, 9]. Inflammation may induce the activation of indoleamine 2,3‐dioxygenase, which catalyzes the degradation of tryptophan into kynurenine. Consequently, the level of brain serotonin is reduced, which leads to depression‐like symptoms [4, 5, 8, 9]. Hence, drugs that modulate brain serotonin function have been used as antidepressants. Fluoxetine (FLX) is one of the most prescribed selective serotonin reuptake inhibitors for depression, which could alleviate depressive symptoms and provide a neuroprotective effect by reducing neuroinflammation [8, 9, 10].

Accumulating evidence indicates that months or even years of psychosocial stress, especially lower socioeconomic status and perceived discrimination, are associated with increased risk of acute kidney injury and chronic kidney disease (CKD) progression in human beings across races [11, 12, 13, 14, 15, 16, 17, 18, 19, 20, 21, 22]. In rodents, immobilization of rats 2 h per day for 5 ~ 6 weeks leads to glomerular loss [23] and chronic psychosocial stress induces interstitial nephritis in CBA mice [24]. Such phenomena can be reproduced in NZM2410/J mice which spontaneously develop lupus nephritis [25]. Social defeat stress led to increased deposits of C3 and IgG complexes and macrophage infiltration in the kidney as well as increased serum levels of inflammatory cytokines and anti‐dsDNA autoantibodies [25]. Therefore, rodents can be used to analyze the mechanisms underlying the association between chronic psychological stress and the deterioration of kidney function. Indeed, it has been shown that experimental depression induces oxidative stress, altered expression of parathormone receptor and tryptophan hydroxylase, and the rate‐limiting enzyme in serotonin synthesis, in the kidney of rats [26, 27, 28]. In this scenario, research into a genome‐wide profile of stress‐induced changes in the renal transcriptome that are associated with the development of depressive behaviors is needed. Furthermore, it is of interest to explore the effects of serotonin reuptake inhibitor on stress‐induced changes in the kidney.

To answer these questions, we constructed chronic social defeat stress (CSDS) model in C57 BL/6 male mice. Compared with other rodent models of depression, this model better mimics the psychosocial stressors, which are inversely associated with kidney function and effectively reproduces the neuropathological and behavioral phenotypes observed in human depression [29]. We aim to provide a genome‐wide view of psychosocial stress‐triggered changes in renal transcriptome and clarify the effects of selective serotonin reuptake inhibitor (SSRI) on stress‐induced changes in the kidney.

## Materials and methods

### Experimental animals

Adult (retired breeders) male CD‐1 mice and 6‐week‐old male C57BL/6 mice were brought from SPF Biotechnology Co., Ltd (Beijing, China). All mice were maintained under specific pathogen‐free conditions with controlled temperature (24 ± 1 °C) and humidity (50 ± 10%) and a 12‐h‐light: 12‐h‐dark cycle. Mice were acclimatized through 7 days of adaptive feeding. Animal care and experiments were performed in strict accordance with the Guide for the Care and Use of Laboratory Animals (National Institutes of Health publication 86‐23, revised 1985) and were approved by the ethics committee of the Beijing Institute of Basic Medical Sciences (Permit number: AMMS2020‐0356).

### CSDS model

**Fig. 1:**
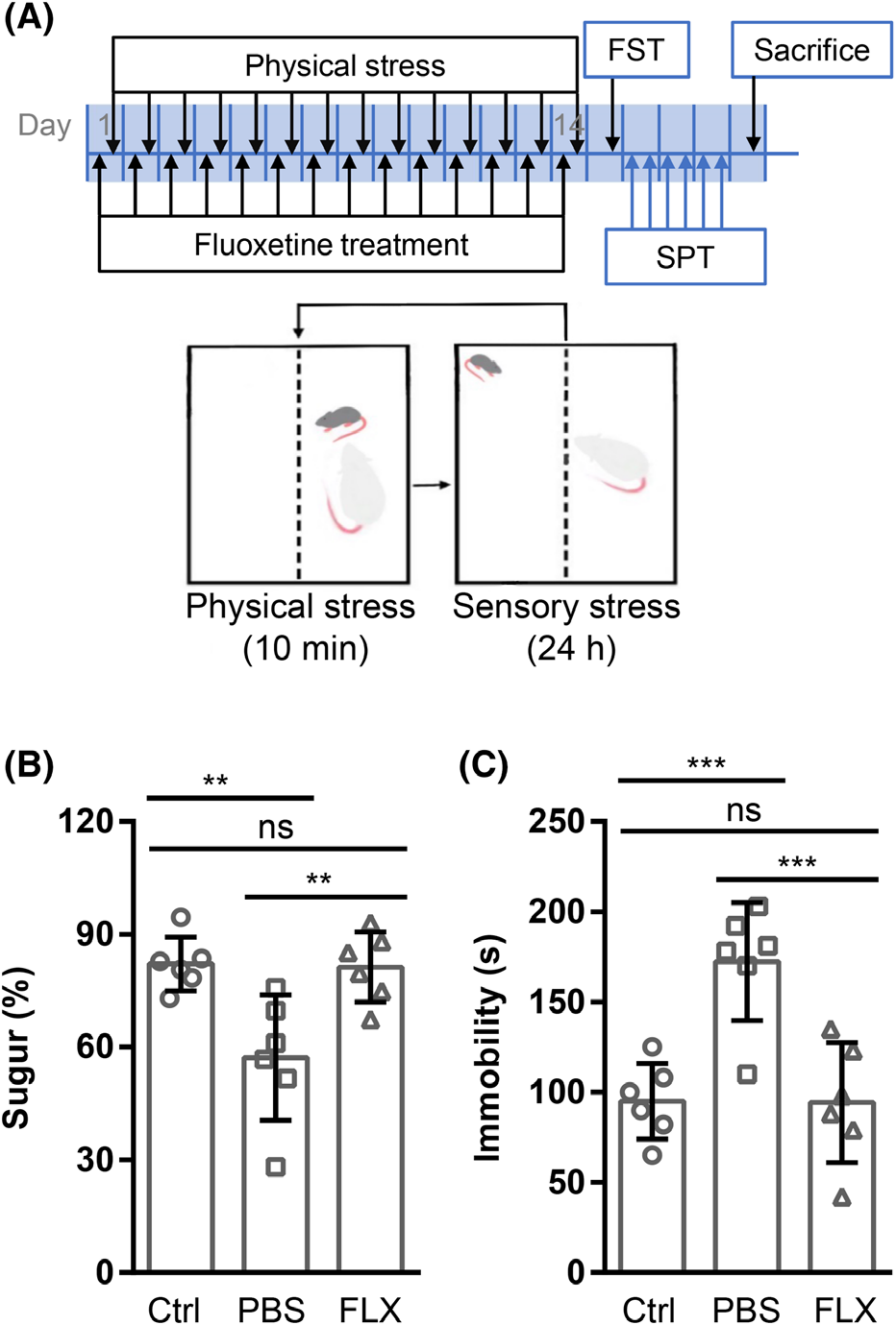
FLX reverses CSDS‐induced depression‐like behaviors. (A) The experiment schedule. (B, C) Six‐week‐old male C57BL/6 mice subjected to CSDS were intraperitoneally injected with FLX (10 mg·kg^−1^) or PBS of equal volume once a day during chronic stress induction. Then, sucrose preference rate (B) and immobility time in forced swim test (C) were measured. Symbols are values from individual mice (*n* = 6/group). The results are shown as mean ± standard deviations and were analyzed using prism 6.0 (GraphPad) by one‐way ANOVA with Dunnett's *post hoc* analysis. **P < 0.01; ***P < 0.001; ns, not significant.

CD‐1 mice were individually housed and strictly screened for 3 consecutive days. CD‐1 mice attacking C57BL/6 mice for at least 2 consecutive days and initiating attack latency ≤30 s were selected for modeling. Male C57BL/6 mice were randomly divided into three groups (*n* = 6/group): control (Ctrl), CSDS + phosphate‐buffered saline (PBS), and CSDS + fluoxetine (FLX). Special cages with a perforated Plexiglas plate separating the space into two halves were used. An intruder C57BL/6 mouse in PBS group or FLX group was directly exposed to a resident CD‐1 mouse for 10 min each day. If the CD‐1 mouse kept continuous biting even after the C57 BL/6 displayed submissive posture, the defeat bout was immediately terminated. At the end of the frustration, the defeated C57BL/6 mouse was transferred to the other side of the cage so that the two mice could still maintain sensory and olfactory contact [29]. This procedure was repeated for 2 weeks with individual C57BL/6 mouse exposed to different CD‐1 mice each day. C57 BL/6 mice in the control group were handled daily and housed in the same type of cages. C57 BL/6 mice in FLX group were intraperitoneally injected once a day with 10 mg·kg^−1^ FLX (Cat# 064‐04323, Wako Chemical Co. Ltd, Tokyo, Japan) dissolved in PBS; at this dose, this selective serotonin reuptake inhibitor can normalize CSDS phenotypes [30, 31], while mice in PBS group injected with the same volume of PBS, during CSDS induction (Fig. 1A).

### Forced swim test (FST)

Forced swim test of C57 BL/6 mice was performed as described previously [30, 31, 32]. Briefly, each C57 BL/6 mouse was put into a cylindrical glass tank with the height of 50 cm and the diameter of 20 cm. The cylindrical tank was cleaned before each mouse was tested. Then, it was filled with tap water which was kept at 23–25 °C. The water level is 40 cm above the bottom to ensure that the mouse's tail or feet cannot touch the bottom of the tank. Each mouse was put in the tank for 6 min. The criterion for immobility is that the mouse stops struggling in the water and does nothing but balance its body by the necessary movements to keep its nose out of the water. The accumulated immobility time of mice in the last 4 min was recorded.

### Sucrose preference test (SPT)

In this experiment, each mouse was individually housed in its own cage, with free access to food and water. During the first 48 h, the C57 BL/6 mice were habituated to drink from two bottles, one filled with 1% sucrose solution and the other contained tap water. The positions of sucrose solution and the tap water were exchanged every 12 h and counterbalanced across the different cages. On the third day, water and sucrose consumption were measured. The sucrose preference was calculated by the following equation [21]: total liquid intake (g) = sucrose solution intake (g) + tap water intake (g); preference = [sucrose solution intake (g)/total liquid intake (g)] × 100%.

### Histology and immunohistochemistry (IHC)

The mice were anesthetized with pentobarbital sodium and perfused by PBS and 4% paraformaldehyde sequentially. Then, kidneys were removed from mice and fixed in 10% buffered formalin for at least 24 h, dehydrated, and infiltrated with paraffin. Five micrometers paraffin sections were then prepared and stained with hematoxylin and eosin (H & E) for brightfield microscopy. IHC was performed using standard protocols with citrate buffer (pH 6.0) pretreatment. Briefly, formaldehyde‐fixed and paraffin‐embedded kidney sections were incubated with primary antibodies at 4 °C overnight and then with horseradish peroxidase‐conjugated secondary antibodies at 37 °C for 30 min. The sections were finally incubated with diaminobenzidine and counterstained with hematoxylin for detection. The antibody against F4/80 was ordered from BD Bioscience (Cat# 610178, San Jose, CA, USA). Goat anti‐mouse IgG was obtained from Jackson Immunoresearch (Cat# 115‐005‐003, West Grove, PA, USA).

### Bulk RNA sequencing

Total RNA was extracted from kidneys with TRIzol reagent (Cat# 15596026, Life Technologies, Carlsbad, CA, USA). The RNA amount and purity of each sample were quantified using NanoDrop ND‐1000 (NanoDrop, Wilmington, DE, USA). The RNA integrity was assessed by Bioanalyzer 2100 (Agilent, Santa Clara, CA, USA) with RIN number > 7.0 and confirmed by electrophoresis with denaturing agarose gel. Poly (A) RNA was purified from 1 μg total RNA using Dynabeads Oligo (dT) (Cat# 25‐61005, Thermo Fisher Scientific, Waltham, MA, USA) for two rounds. Then, poly (A) RNA was fragmented into small pieces using Magnesium RNA Fragmentation Module (Cat# E6150, New England Biolabs, Herts, UK) at 94 °C for 5–7 min. The cleaved RNA fragments were reverse‐transcribed to create cDNAs by SuperScript™ II Reverse Transcriptase (Cat#1896649, Invitrogen, Carlsbad, CA, USA), which were next used to synthesize U‐labeled second‐stranded DNAs with *E. coli* DNA polymerase I (Cat# M0209, New England Biolabs), RNase H (Cat# M0297, New England Biolabs), and dUTP Solution (Cat# R0133, Thermo Fisher Scientific). An A‐base was then added to the blunt ends of each strand, preparing them for ligation to the indexed adaptors. Each adaptor contained a T‐base overhang for ligating the adaptor to the A‐tailed fragmented DNA. Single‐ or dual‐index adaptors were ligated to the fragments, and size selection was performed with AMPure XP beads (Cat# A63881, Beckman Coulter, Bria, CA, USA). After the heat‐labile UDG enzyme (Cat# M0280, New England Biolabs) treatment of the U‐labeled second‐stranded DNAs, the ligated products were amplified with PCR by the following conditions: initial denaturation at 95 °C for 3 min; 8 cycles of denaturation at 98 °C for 15 s, annealing at 60 °C for 15 s, and extension at 72 °C for 30 s; and then final extension at 72 °C for 5 min. The average insert size for the final cDNA library was 300 ± 50 bp. Finally, we performed the 2 × 150‐bp paired‐end sequencing (PE150) on an Illumina Novaseq™ 6000 (LC‐Bio Technology CO., Ltd., Hangzhou, China) following the vendor's recommended protocol.

### Analysis of bulk RNA‐sequencing data

We used fastp software (v2.2.0) (Haplox, Shenzhen, GuangZhou Province, China) to remove the reads that contained adaptor contamination, low‐quality bases, and undetermined bases with default parameters. Then, sequence quality was also verified using fastp [33]. We used HISAT‐3N beta to map reads to the GRCm38 mouse reference genome [34]. The mapped reads of each sample were assembled using StringTie with default parameters. Then, all transcriptomes from all samples were merged to reconstruct a comprehensive transcriptome using the gffcompare program inside stringtie (the Center for Computational Biology, Johns Hopkins University, MD, USA). After the final transcriptome was generated, StringTie was used to estimate the expression levels of all transcripts by calculating FPKM [total_exon_fragments/mapped_reads (millions) × exon_length (kB)].

### Enrichment analysis

For the differentially expressed genes between different groups (|log_2_FoldChange| > 1 and *P*‐value < 0.05), gene set enrichment analysis (GSEA) was conducted to investigate the biological process by the r package clusterprofiler [35, 36]. The Gene Ontology (GO) biological process (BP) gene sets were downloaded from the Molecular Signature database (https://www.gsea‐msigdb.org/gsea/msigdb) [37]. *P‐*value < 0.05 was set as the cutoff criterion to screen the prominent biological processes.

### Statistical analysis

Quantitative data are shown as mean ± standard deviations and were analyzed using prism 6.0 (GraphPad, Boston, MA, USA). One‐way ANOVA with Dunnett's *post hoc* analysis was used to evaluate quantitative variables. *P* < 0.05 was considered significant.

## Results

### FLX reverses CSDS‐induced depression‐like behaviors

Fourteen days of repeated defeats led to anhedonia as reflected by the sucrose preference test (Fig. 1B, *P* = 0.0043) and psychomotor retardation as reflected by forced swim test (Fig. 1C, *P* = 0.0008). FLX reversed CSDS‐induced anhedonia as reflected by sucrose preference test (Fig. 1B, *P* = 0.0054). FLX also reversed CSDS‐induced psychomotor retardation as reflected by the forced swim test (Fig. 1C, *P* = 0.0007).

### CSDS fails to induce overt renal inflammation

**Fig. 2:**
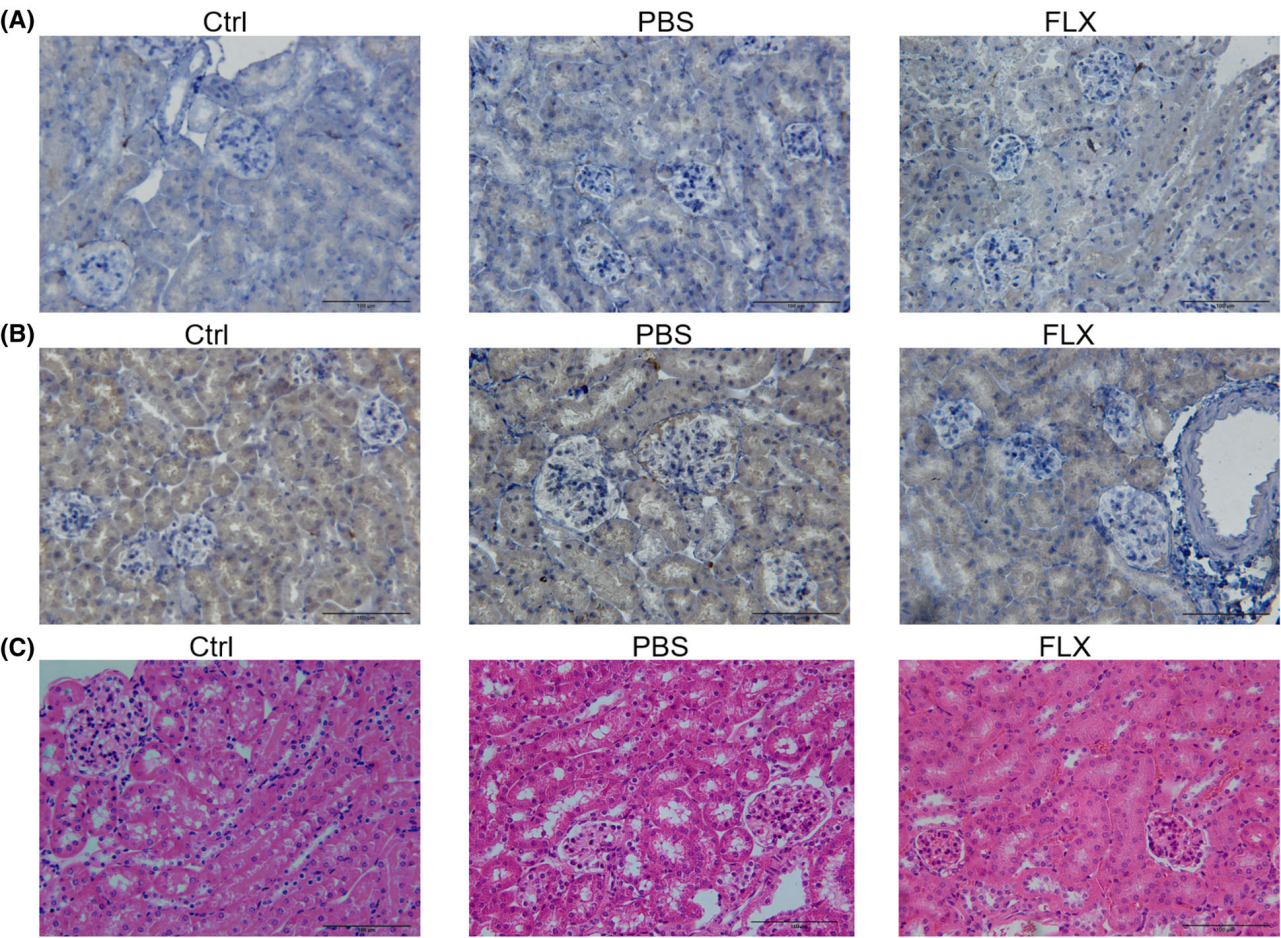
CSDS fails to induce overt renal inflammation. (A–C) The mice in the three groups (Ctrl, PBS, and FLX) were sacrificed and the left kidneys tissues were removed to be subjected to immunohistochemistry (IHC) of F4/80 (A), IgG (B), and H & E staining (C). Scale bar, 100 μm.

We then analyzed whether CSDS induces macrophage infiltration and IgG deposits in the kidney of male C57 BL/6 mice. Immunohistochemistry revealed no F4/80‐positive cells infiltration under CSDS (Fig. 2A). Kidney tubules were IgG‐positive even under steady‐state conditions (Fig. 2B). However, CSDS showed no effects on IgG immunohistochemical staining (Fig. 2B). Accordingly, CSDS showed no visible effects on the histology of the kidney (Fig. 2C).

### CSDS changes the renal transcriptome

**Fig. 3:**
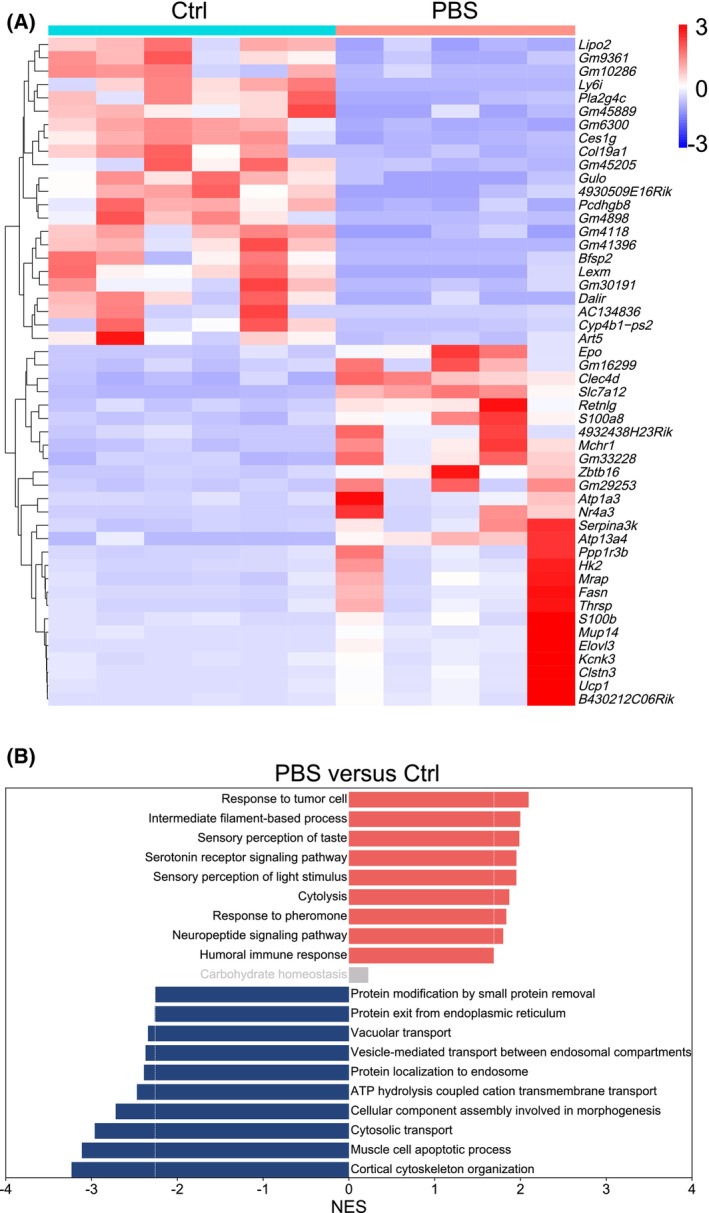
CSDS changes the renal transcriptome. (A, B) The mice in the three groups (Ctrl and PBS, and FLX) were sacrificed, and the right kidneys were removed to be subjected to bulk RNA sequencing. Heatmap of differentially expressed genes between Ctrl and PBS groups (A) and the corresponding enriched GO_BP terms by GSEA (B) are shown. Ctrl group: *n* = 6; PBS group: *n* = 5.

We then employed bulk RNA‐sequencing analysis to explore whether CSDS has some detrimental effects on the kidney of C57 BL/6 male and whether all the detrimental effects could be reversed by FLX treatment. The genes were deemed to be differentially expressed if |log_2_FoldChange| > 1 and *P*‐value < 0.05. The data of a mouse in PBS group were too different from others with excessive expression of inflammatory factors and therefore were deleted before further analysis. As expected, CSDS changed the kidney transcriptome with 398 up‐regulated genes and 391 down‐regulated, among which the top 50 differentially expressed genes are shown (Fig. 3A). To explore the effects of CSDS, GSEA was used to show the biological process of CSDS. The results showed that the CSDS up‐regulated genes were involved in processes such as serotonin receptor signaling pathway (*P* = 0.0135), neuropeptide signaling pathway (*P* = 0.0318), response to pheromone (*P* = 0.0181), cytolysis (*P* = 0.0262), and humoral immune response (*P* = 0.0220; Fig. 3B). Meanwhile, the down‐regulated genes were involved in cytosolic transport (*P* < 0.0001), muscle cell apoptotic process (*P* < 0.0001), and cellular component assembly process involved in morphogenesis (*P* < 0.0001).

### FLX treatment changes the renal transcriptome under CSDS

**Fig. 4:**
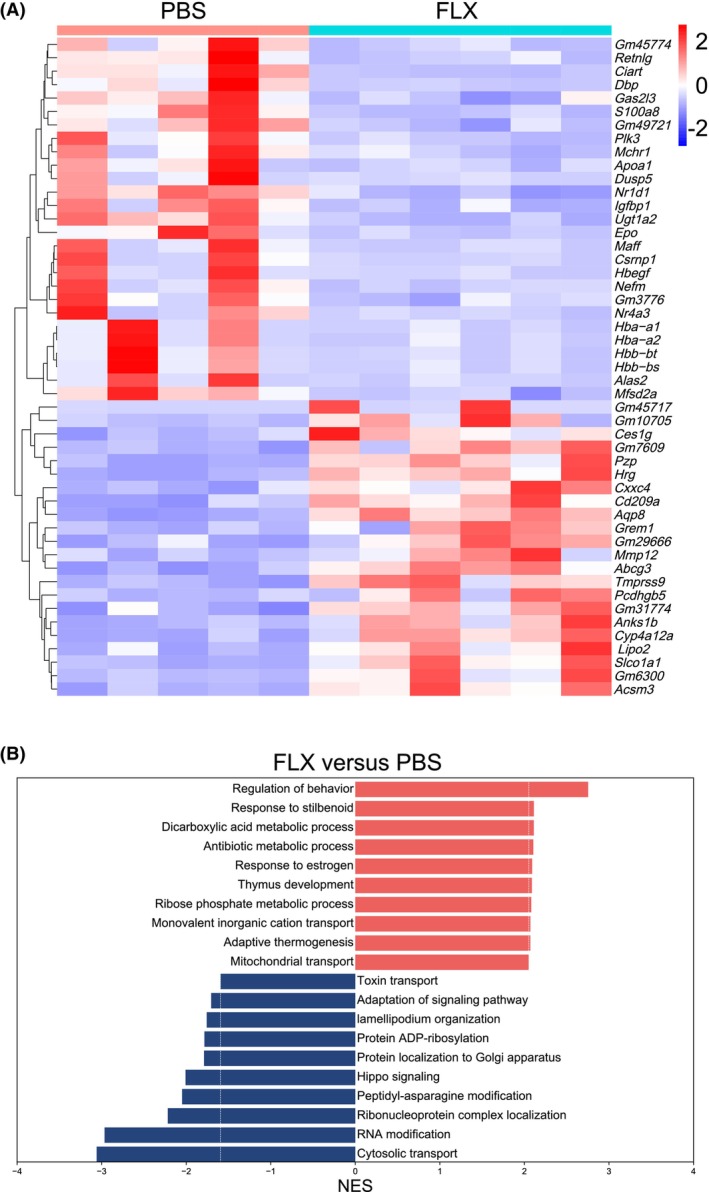
FLX treatment changes the renal transcriptome under CSDS. (A, B) The mice in the three groups (Ctrl and PBS, FLX) were sacrificed and the right kidneys were removed to be subjected to bulk RNA sequencing. Heatmap of differentially expressed genes between PBS and FLX groups (A) and the corresponding enriched GO_BP terms by GSEA (B) are shown. PBS group: *n* = 5; FLX group: *n* = 6.

Next, we analyzed how FLX treatment changes the transcriptome in the kidney of male C57 BL/6 mice. Bulk RNA‐sequencing analysis revealed that FLX treatment changed the kidney transcriptome under the condition of CSDS with 211 up‐regulated genes and 222 down‐regulated genes, among which the top 50 differentially expressed genes were shown (Fig. 4A). GSEA analysis revealed that FLX treatment down‐regulated genes involved in the processes such as RNA modification (*P* < 0.0001), cytosolic transport (*P* < 0.0001), hippo signaling (*P* < 0.0001), and peptidyl‐asparagine modification (*P* = 0.0066). Meanwhile, FLX treatment also up‐regulated genes involved in the processes such as mitochondrial transport (*P* < 0.0001), adaptive thermogenesis (*P* = 0.0010), thymus development (*P* = 0.0042), and dicarboxylic acid metabolic process (*P* < 0.0001; Fig. 4B).

### FLX treatment partially reverses the effects of CSDS on renal gene expression

**Fig. 5:**
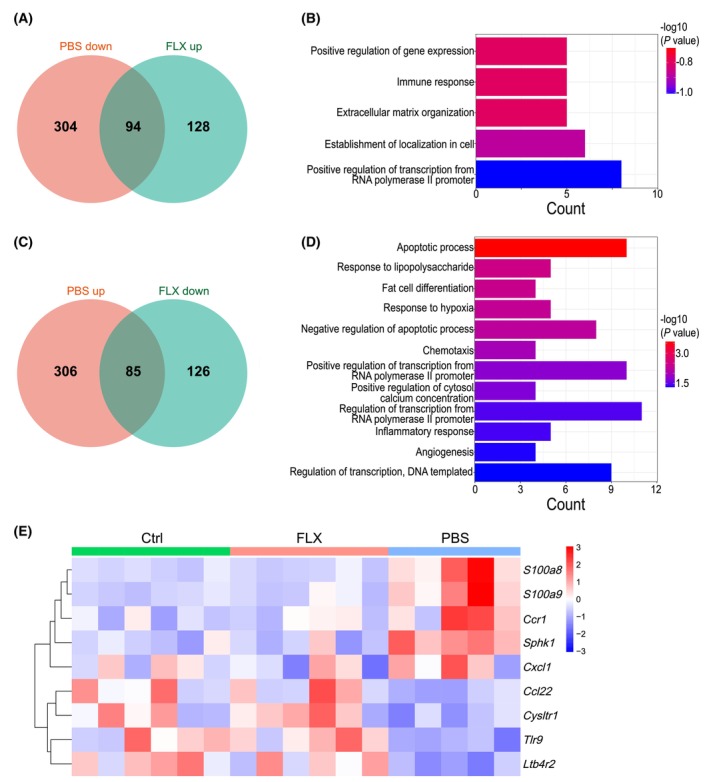
FLX treatment partially reverses the effects of CSDS on renal gene expression. (A–E) The mice in the three groups (Ctrl and PBS, FLX) were sacrificed and the right kidneys were removed to be subjected to bulk RNA sequencing. Venn diagram of the common genes up‐regulated in FLX group while down‐regulated in PBS group (A) or down‐regulated in FLX group while up‐regulated in PBS group (C). GO_BP terms of common genes up‐regulated in FLX group while down‐regulated in PBS group (B) or down‐regulated in FLX while up‐regulated in PBS group (D). Heatmap of genes associated with inflammation (E). Ctrl group: *n* = 6; PBS group: *n* = 5; FLX group: *n* = 6.

Finally, we compared the transcriptome of the three groups. The results showed that the down‐regulated genes of CSDS and the up‐regulated ones of FLX treatment shared 94 genes in common (Fig. 5A), while the up‐regulated of CSDS and the down‐regulated of FLX treatment shared 85 in common (Fig. 5C). The GO_BP enrichment analysis showed that these genes are involved in the processes such as inflammatory response (*P* = 0.0393, Fig. 5D), immune response (*P* = 0.0299, Fig. 5B), chemotaxis (*P* = 0.0102, Fig. 5D), and apoptotic process (*P* = 0.0001, Fig. 5D). And the inflammation‐related genes are shown in Fig. 5E, including the genes involved in inducing renal inflammation, such as *S100a8*, (PBS versus Ctrl: *P* = 0.0001; FLX versus PBS: *P* < 0.0001), *S100a9* (PBS versus Ctrl: *P* < 0.0001; FLX versus PBS: *P* = 0.0041), *Ccr1* (PBS versus Ctrl: *P* = 0.0027; FLX versus PBS: *P* = 0.0065), *Sphk1* (PBS versus Ctrl: *P* < 0.0001; FLX versus PBS: *P* < 0.0001), and *Cxcl1* (PBS versus Ctrl: *P* = 0.0086; FLX versus PBS: *P* = 0.0085). These results suggest that FLX could alleviate the inflammatory effects of CSDS in the kidney of male C57 BL/6 mice through regulation of the gene expression.

### The kidney expresses receptors for stress hormones and serotonin

**Fig. 6:**
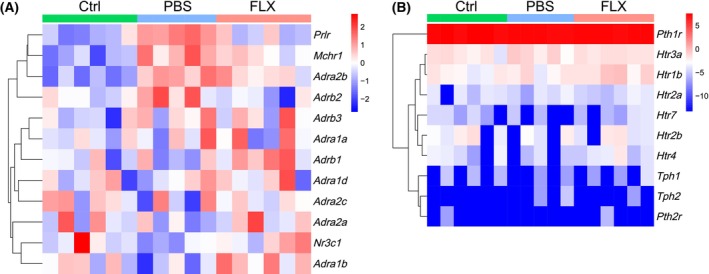
Kidney expresses receptors for stress hormones and serotonin. (A, B) The mice in the three groups (Ctrl and PBS, FLX) were sacrificed and the right kidneys were removed to be subjected to bulk RNA sequencing. The expression of receptors for stress hormones (A) and serotonin (B) in Ctrl, PBS, and FLX groups is shown. Ctrl group: *n* = 6; PBS group: *n* = 5; FLX group: *n* = 6.

The molecular basis for the aforementioned changes in the kidney transcriptome was also evaluated. We tried to explore the expression of stress hormone receptors as well as serotonin receptors. Transcripts of glucocorticoid receptor gene *Nr3c1* and adrenergic receptor genes *Adra1a*, *Adra1b*, *Adra1d*, *Adra2a*, *Adra2b*, *Adra2c*, *Adrb1*, *Adrb2*, *and Adrb3* were detected in the kidney of male C57 BL/6 mice, which were not changed by CSDS with or without FLX treatment (Fig. 6A). Transcripts of prolactin receptor gene *Prlr* (PBS versus Ctrl: *P* < 0.0001; FLX versus PBS: *P* < 0.0001) and melanin‐concentrating hormone receptor *Mchr1* (PBS versus Ctrl: *P* < 0.0001; FLX versus PBS: *P* = 0.0001) were also detected, which were both up‐regulated by CSDS and reversed by FLX treatment (Fig. 6A). On the contrary, transcripts of serotonin receptor genes *Htr1b*, *Htr2a*, *Htr2b*, *Htr3a*, *Htr4*, and *Htr7* were also detected, which were not changed by CSDS (Fig. 6B). In addition, the expression of parathormone receptor genes (*Pth1r* and *Pth2r*) and tryptophan hydroxylase genes (*Tph1* and *Tph2*), was changed insignificantly in the defeated male C57 BL/6 mice (Fig. 6B).

## Discussion

The present study provides a genome‐wide view of CSDS‐triggered changes in renal transcriptome. FLX treatment changes the renal transcriptome under the condition of CSDS, which partially reverses the effects of CSDS on renal gene expression and has additional effects. The molecular basis for these changes might be attributed, at least partially, to the expression of receptors for stress hormones and serotonin in the kidney.

CSDS led to depression‐like behaviors in male C57 BL/6 mice (Fig. 1B,C) with 398 up‐regulated genes and 391 down‐regulated in the kidney (Fig. 3A). Recently, it has been revealed that most of the differentially expressed genes in the prefrontal cortex of depressed patients are immediate early genes such as *Nr4a3* [38]. In line with these findings, several immediate early genes such as *Nr4a3* were up‐regulated in the kidney of defeated male C57 BL/6 mice (Fig. 3A). In the brain of male mice after CSDS, it has been demonstrated that the most reproducible transcriptome changes are the expression of hemoglobin genes [38, 39], which is believed to provide neuroprotection in response to oxidative stress [40]. Intriguingly, the expression of hemoglobin *genes Hba‐a1*, *Hba‐a2*, *Hbb‐bs*, and *Hbb‐bt* was detected in the kidney of male C57 BL/6 mice and showed a trend of up‐regulation after CSDS although it failed to reach statistical difference (data not shown). These data are somewhat consistent with the reported oxidative stress in the kidney of rats with experimental depression [26]. In addition, tryptophan hydroxylase protein level was reported to be reduced in the kidney of rats with experimental depression [28]. Accordingly, tryptophan hydroxylase transcripts were detected in the kidney of male C57 BL/6 mice although the effect of CSDS was not significant (Fig. 6B). Furthermore, transcripts of serotonin receptor genes were also detected (Fig. 6B), which can explain the enriched serotonin receptor signaling pathway after CSDS. Besides, the enrichment of CSDS down‐regulated genes highlighted cellular transport and cell component assembly process which consume energy (Fig. 3B). Thus, the above changes might reflect the cellular adaptability to cope with the detrimental effects of CSDS on the kidney. However, the cellular adaption seems not enough because the enrichment of CSDS up‐regulated genes also highlighted cytolysis (Fig. 3B). Furthermore, the expression of several inflammation‐related genes was altered by CSDS (Fig. 5E). Among these genes, *S100a8*, *S100a9*, *Ccr1*, *Sphk1*, and *Cxcl1* have been reported to be involved in inducing renal inflammation [41, 42, 43, 44]. The results show that CSDS leads to an inflammatory transcriptome, even though with no overt effects on the kidney of male C57 BL/6 mice. In line with a previous report [45], the kidney tubules were IgG‐positive even under steady‐state conditions (Fig. 2B). However, CSDS showed no effects on IgG immunohistochemical staining (Fig. 2B). Accordingly, CSDS showed no visible effects on the histology of the kidney (Fig. 2C).

In the present work, transcripts of glucocorticoid receptor gene, adrenergic receptor genes, prolactin receptor gene, and melanin‐concentrating hormone receptor were detected in the kidney of male C57 BL/6 mice (Fig. 6A). Therefore, CSDS might exert detrimental effects on the kidney directly through stress hormones and their receptors. Accordingly, the enrichment of CSDS down‐regulated genes highlighted neuropeptide signaling pathway and response to pheromone (Fig. 3B). Furthermore, it has been reported that chronic mild stress leads to increased expression of *Prlr* and *Mchr1* [46, 47]. Indeed, CSDS‐induced up‐regulation of *Prlr* and *Mchr1* expression (Fig. 6A). Psychological stress‐induced release of prolactin promotes pro‐inflammatory immune responses via nuclear factor‐κB and interferon regulatory factor‐1 [48, 49]. On the contrary, melanin‐concentrating hormone, also up‐regulated by psychological stress, has been implicated in the regulation of feeding, emotional processing, and sleep in rodents [50]. And antagonists of melanin‐concentrating hormone receptor produce strong antidepressant and antianxiety effects in various models of depression and anxiety [47, 50, 51]. In addition, it has been reported that CSDS could shift renal circadian clock phase by affecting renal clock gene expression in male C57 BL/6 mice, and the detailed mechanism might be that stress and stress hormones could exert potent effects on peripheral clocks [52]. Indeed, the clock genes *Per1* and *Cry1* were up‐regulated by CSDS (data not shown), which might promote the activation of nuclear factor‐κB [53]. Therefore, CSDS exerts detrimental effects on the kidney through various mechanisms.

Fluoxetine treatment reversed CSDS‐induced depression‐like behaviors (Fig. 1B,C) and partially reversed the effects of CSDS on renal gene expression (Fig. 5A–E). It has been reported that immediate early genes are the most prominent transcripts modulated by FLX during the induction of depression in different animal models [54]. In line with these observations, the up‐regulation of immediate early genes such as *Nr4a3* in the kidney of defeated male C57 BL/6 mice was reversed by FLX (Fig. 4A). Recently, a protective role of FLX against oxidative stress in the kidney has been revealed [55]. Accordingly, FLX treatment led to reduced expression of hemoglobin genes in the kidney of defeated male C57 BL/6 mice (Fig. 4A). Furthermore, FLX treatment reversed the aberrant expression of several inflammation‐related genes in the kidney of defeated male C57 BL/6 mice (Fig. 5E), which is consistent with the reported anti‐inflammatory role of FLX [56, 57].

Fluoxetine treatment has been reported to reduce the levels of serum stress hormones such as cortisol and melanin‐concentrating hormone [57, 58, 59], which might alleviate the detrimental effects of CSDS on the kidney. On the contrary, kidney of male C57 BL/6 mice express tryptophan hydroxylase (Fig. 6B), the rate‐limiting enzyme in serotonin synthesis [28], so FLX treatment might lead to local serotonin accumulation. Importantly, the kidney of male C57 BL/6 mice express serotonin receptors. Therefore, locally accumulated serotonin can directly regulate gene expression in kidney tissues. Moreover, it has been demonstrated that FLX treatment can reverse chronic mild stress‐induced up‐regulation of *Mchr1* [49]. The present study shows that FLX treatment reversed CSDS‐induced up‐regulation of both *Prlr* and *Mchr1* (Fig. 6A). In addition, FLX treatment has been revealed to down‐regulated clock gene *Per1* [60]. Indeed, FLX treatment reversed CSDS‐induced up‐regulation of both *Per1* and *Cry1* (data not shown). Therefore, FLX treatment can alleviate the detrimental effects of depression through multiple mechanisms.

The present work has some limitations. First, the sample size for behavioral research is small. Since the CSDS model can cause resilience in a small portion of animals [29], it would be better if *n* ≥ 8. Second, even though the results of the behavior tests FST and SPT verified the facticity of depression, social avoidance, a classical manifestation of depression, and tail suspension test, another indicator of psychomotor retardation, should be assayed. Third, the CSDS model did not take gender effects into consideration. As is known to all, women are prone to be affected by depression than men, because chromosomal and environmental factors contribute to sex differences in vulnerability to depression [61]. Fourth, there is no stress‐free group receiving FLX. Although there is shared signature of FLX in stressed and naïve rodents, transcriptomic alterations after FLX treatment in naïve mice or in mice subjected to stress‐induced models of depression are quite different [54]. Even though FLX shows a well‐established safety profile [62], it might affect kidney potassium channels and perinatal kidney development [63, 64]. Without a stress‐free group receiving FLX, there is no way to adjudge how FLX might exert nephrotoxicity by affecting gene expression. Finally, ineluctable wounds during the CSDS process should be avoided, which might affect the results of inflammatory response.

Future studies should employ more rodent models of psychological stress and larger sample size to explore whether there are some differences in stress‐induced changes in the renal transcriptome between male and female rodents. The establishment of CSDS models in female mice by recent studies [65, 66] facilitates achieving these goals. Besides FLX, other antidepressants can be used to explore their potential application to alleviate kidney damage while improving mood. It is important to include a stress‐free group receiving the corresponding antidepressant(s). The corresponding findings can incite clinical translational research, for example, a randomized controlled trial about an antidepressant in CKD patients. Furthermore, the present study has identified many differentially expressed genes and enriched pathways, their roles in kidney function and the molecular mechanisms underlying their dynamic expression remain to be explored.

## Conclusion

In this work, we propose that chronic psychosocial stress induces inflammatory changes in the kidney, and this could be reversed partially by FLX treatment. The detrimental effects of chronic psychosocial stress might persist and eventually aggravate acute kidney injury or accelerate CKD progression. Antidepressants can be used to alleviate kidney damage while improving mood.

## Conflict of interest

The authors declare no conflict of interest.

### Peer review

The peer review history for this article is available at https://www.webofscience.com/api/gateway/wos/peer‐review/10.1002/2211‐5463.13670.

## Author contributions

HJ and JZ conceived and designed the project. GX, YL, CN, XZ, JC, XY, and QC acquired the data. YL, TK, JZ, and JD analyzed and interpreted the data. YL, JZ, and JD wrote the original draft. All authors discussed the results and approved the final manuscript.

## Data Availability

Bulk RNA‐sequencing data that support the findings in this study are openly available in NCBI's Gene Expression Omnibus and are accessible through https://www.ncbi.nlm.nih.gov/geo/query/acc.cgi?acc=GSE207710, GEO Series accession number [GSE207710], reviewer token cdydeuwuflwblgx.

## References

[1] World Health Organization (2022) World mental health report: transforming mental health for all. Available at: https://www.who.int/publications/i/item/9789240049338.

[2] GDB (2019) Mental disorders collaborators (2022) global, regional, and national burden of 12 mental disorders in 204 countries and territories, 1990‐2019: a systematic analysis for the global burden of disease study 2019. Lancet Psychiatry 9, 137–150. 10.1016/S2215-0366(21)00395-3 PMC8776563 35026139

[3] Smith K (2014) Mental health: a world of depression. Nature 515, 181. 25391942 10.1038/515180a

[4] Wang H , He Y , Sun Z , Ren S , Liu M , Wang G and Yang J (2022) Microglia in depression: an overview of microglia in the pathogenesis and treatment of depression. J Neuroinflammation 19, 132. 35668399 10.1186/s12974-022-02492-0 PMC9168645

[5] Hersey M , Hashemi P and Reagan LP (2022) Integrating the monoamine and cytokine hypotheses of depression: is histamine the missing link? Eur J Neurosci 55, 2895–2911. 34265868 10.1111/ejn.15392

[6] Dhabhar FS (2014) Effects of stress on immune function: the good, the bad, and the beautiful. Immunol Res 58, 193–210. 24798553 10.1007/s12026-014-8517-0

[7] Furman D , Campisi J , Verdin E , Carrera‐Bastos P , Tang S , Franceschi C , Ferrucci L , Gilroy DW , Fasano A , Miller GW *et al*. (2019) Chronic inflammation in the etiology of disease across the life span. Nat Med 25, 1822–1832. 31806905 10.1038/s41591-019-0675-0 PMC7147972

[8] Vahid‐Ansari F and Albert PR (2021) Rewiring of the serotonin system in major depression. Front Psych 12, 802581. 10.3389/fpsyt.2021.802581 PMC8716791 34975594

[9] Fluyau D , Mitra P , Jain A , Kailasam VK and Pierre CG (2022) Selective serotonin reuptake inhibitors in the treatment of depression, anxiety, and post‐traumatic stress disorder in substance use disorders: a Bayesian meta‐analysis. Eur J Clin Pharmacol 78, 931–942. 35246699 10.1007/s00228-022-03303-4

[10] Kryst J , Majcher‐Maslanka I and Chocyk A (2022) Effects of chronic fluoxetine treatment on anxiety‐ and depressive‐like behaviors in adolescent rodents‐systematic review and meta‐analysis. Pharmacol Rep 74, 920–946. 36151445 10.1007/s43440-022-00420-w PMC9584991

[11] Shoham DA , Vupputuri S , Diez Roux A , Kaufman JS , Coresh J , Kshirsagar A , Zeng D and Heiss G (2007) Kidney disease in life‐course socioeconomic context: the atherosclerosis risk in communities (ARIC) study. Am J Kidney Dis 49, 217–226. 17261424 10.1053/j.ajkd.2006.11.031

[12] Bruce MA , Beech BM , Crook ED , Sims M , Wyatt SB , Flessner MF , Taylor HA , Williams DR , Akylbekova EL and Ikizler TA (2010) Association of socioeconomic status and CKD among African Americans: the Jackson heart study. Am J Kidney Dis 55, 1001–1008. 20381223 10.1053/j.ajkd.2010.01.016 PMC2876216

[13] Crews DC , Charles RF , Evans MK , Zonderman AB and Powe NR (2010) Poverty, race, and CKD in a racially and socioeconomically diverse urban population. Am J Kidney Dis 55, 992–1000. 20207457 10.1053/j.ajkd.2009.12.032 PMC2876201

[14] Beydoun MA , Poggi‐Burke A , Zonderman AB , Rostant OS , Evans MK and Crews DC (2017) Perceived discrimination and longitudinal change in kidney function among urban adults. Psychosom Med 79, 824–834. 28445210 10.1097/PSY.0000000000000478 PMC5595064

[15] Lunyera J , Davenport CA , Bhavsar NA , Sims M , Scialla J , Pendergast J , Hall R , Tyson CC , Clair Russell J , Wang W *et al*. (2018) Nondepressive psychosocial factors and CKD outcomes in black Americans. Clin J Am Soc Nephrol 13, 213–222. 29298761 10.2215/CJN.06430617 PMC5967427

[16] Adjei DN , Stronks K , Adu D , Beune E , Meeks K , Smeeth L , Addo J , Owusu‐Dabo E , Klipstein‐Grobusch K , Mockenhaupt F *et al*. (2019) Cross‐sectional study of association between psychosocial stressors with chronic kidney disease among migrant and non‐migrant Ghanaians living in Europe and Ghana: the RODAM study. BMJ Open 9, e017931. 10.1136/bmjopen-2018-027931 PMC6688695 31375611

[17] Lunyera J , Stanifer JW , Davenport CA , Mohottige D , Bhavsar NA , Scialla JJ , Pendergast J , Boulware LE and Diamantidis CJ (2020) Life course socioeconomic status, allostatic load, and kidney health in black Americans. Clin J Am Soc Nephrol 15, 341–348. 32075808 10.2215/CJN.08430719 PMC7057315

[18] Glover LM , Butler‐Williams C , Cain‐Shields L , Forde AT , Purnell TS , Young B and Sims M (2020) Optimism is associated with chronic kidney disease and rapid kidney function decline among African Americans in the Jackson heart study. J Psychosom Res 139, 110267. 33069050 10.1016/j.jpsychores.2020.110267 PMC7722009

[19] Kim JY , Joo YS , Jhee JH , Han SH , Yoo TH , Kang SW and Park JT (2021) Effect of psychosocial distress on the rate of kidney function decline. J Gen Intern Med 36, 2966–2974. 33469756 10.1007/s11606-020-06573-9 PMC8481510

[20] Su G , Song H , Lanka V , Liu X , Fang F , Valdimarsdóttir UA and Carrero JJ (2021) Stress related disorders and the risk of kidney disease. Kidney Int Rep 6, 706–715. 33732985 10.1016/j.ekir.2020.12.032 PMC7938078

[21] Cain‐Shields L , Glover L , Young B and Sims M (2021) Association between goal‐striving stress and rapid kidney function decline among African Americans: the Jackson heart study. J Invest Med 69, 382–387. 10.1136/jim-2020-001510 PMC8057281 33335024

[22] Koga K , Hara M , Shimanoe C , Nishida Y , Furukawa T , Iwasaka C , Tanaka K , Otonari J , Ikezaki H , Kubo Y *et al*. (2022) Association of perceived stress and coping strategies with the renal function in middle‐aged and older Japanese men and women. Sci Rep 12, 291. 34997128 10.1038/s41598-021-04324-2 PMC8742036

[23] de Souza DB , Silva D , Silva CMC , Sampaio FJB , Costa WS and Cortez CM (2011) Effects of immobilization stress on kidneys of Wistar male rats: a morphometrical and stereological analysis. Kidney Blood Press Res 34, 424–429. 21709423 10.1159/000328331

[24] Henry JP , Meehan WP and Stephens PM (1982) Role of subordination in nephritis of socially stressed mice. Contrib Nephrol 30, 38–42. 6126316 10.1159/000406416

[25] Aqel SI , Hampton JM , Bruss M , Jones KT , Valiente GR , Wu LC , Young MC , Willis WL , Ardoin S , Agarwal S *et al*. (2017) Daily moderate exercise is beneficial and social stress is detrimental to disease pathology in murine lupus nephritis. Front Physiol 8, 236. 28491039 10.3389/fphys.2017.00236 PMC5405126

[26] Pedreanez A , Arcaya JL , Carrizo E , Rincon J , Viera N , Pena C , Vargas R and Mosquera J (2011) Experimental depression induces renal oxidative stress in rats. Physiol Behav 104, 1002–1009. 21741982 10.1016/j.physbeh.2011.06.021

[27] Terzioglu‐Usak S , Elibol B , Dalli T , Guler C and Aysan E (2018) Effect of restraint stress on plasma PTH concentration and its molecular targets expressions in Wistar rats. Int J Endocrinol Metab 16, e66979. 30464774 10.5812/ijem.66979 PMC6216602

[28] Chen Y , Xu H , Zhu M , Liu K , Lin B , Luo R , Chen C and Li M (2017) Stress inhibits tryptophan hydroxylase expression in a rat model of depression. Oncotarget 8, 63247–63257. 28968985 10.18632/oncotarget.18780 PMC5609917

[29] Golden SA , Covington HE 3rd , Berton O and Russo SJ (2011) A standardized protocol for repeated social defeat stress in mice. Nat Protoc 6, 1183–1191. 21799487 10.1038/nprot.2011.361 PMC3220278

[30] Dulawa SC , Holick KA , Gundersen B and Hen R (2004) Effects of chronic fluoxetine in animal models of anxiety and depression. Neuropsychopharmacology 29, 1321–1330. 15085085 10.1038/sj.npp.1300433

[31] Cordner ZA , Khambadkone SG , Zhu S , Bai J , Forti RR , Goodman E , Tamashiro KLK and Ross CA (2021) Ankyrin‐G heterozygous knockout mice display increased sensitivity to social defeat stress. Complex Psychiatry 7, 71–79. 35928299 10.1159/000518819 PMC8740233

[32] Can A , Dao DT , Arad M , Terrillion CE , Piantadosi SC and Gould TD (2012) The mouse forced swim test. J Vis Exp 59, e3638. 10.3791/3638 PMC3353513 22314943

[33] Chen S , Zhou Y , Chen Y and Gu J (2018) Fastp: an ultra‐fast all‐in‐one FASTQ preprocessor. Bioinformatics 34, i884–i890. 30423086 10.1093/bioinformatics/bty560 PMC6129281

[34] Zhang Y , Park C , Bennett C , Thornton M and Kim D (2021) Rapid and accurate alignment of nucleotide conversion sequencing reads with HISAT‐3N. Genome Res 31, 1290–1295. 34103331 10.1101/gr.275193.120 PMC8256862

[35] Subramanian A , Tamayo P , Mootha VK , Mukherjee S , Ebert BL , Gillette MA , Paulovich A , Pomeroy SL , Golub TR , Lander ES *et al*. (2005) Gene set enrichment analysis: a knowledge‐based approach for interpreting genome‐wide expression profiles. Proc Natl Acad Sci USA 102, 15545–15550. 16199517 10.1073/pnas.0506580102 PMC1239896

[36] Yu G , Wang LG , Han Y and He QY (2012) clusterProfiler: an R package for comparing biological themes among gene clusters. OMICS 16, 284–287. 22455463 10.1089/omi.2011.0118 PMC3339379

[37] Liberzon A , Subramanian A , Pinchback R , Thorvaldsdottir H , Tamayo P and Mesirov JP (2011) Molecular signatures database (MSigDB) 3.0. Bioinformatics 27, 1739–1740. 21546393 10.1093/bioinformatics/btr260 PMC3106198

[38] Reshetnikov VV , Kisaretova PE and Bondar NP (2022) Transcriptome alterations caused by social defeat stress of various durations in mice and its relevance to depression and posttraumatic stress disorder in humans: a meta‐analysis. Int J Mol Sci 23, 13792. 36430271 10.3390/ijms232213792 PMC9698544

[39] Vennin C , Hewel C , Todorov H , Wendelmuth M , Radyushkin K , Heimbach A , Horenko I , Ayash S , Muller MB , Schweiger S *et al*. (2022) A resilience related glial‐neurovascular network is transcriptionally activated after chronic social defeat in male mice. Cell 11, 3405. 10.3390/cells11213405 PMC9655779 36359800

[40] Cabezas R , Baez‐Jurado E , Hidalgo‐Lanussa O , Echeverria V , Ashrad GM , Sahebkar A and Barreto GE (2019) Growth factors and neuroglobin in astrocyte protection against neurodegeneration and oxidative stress. Mol Neurobiol 56, 2339–2351. 29982985 10.1007/s12035-018-1203-9

[41] Tesch G , Sourris KC , Summers SA , McCarthy D , Ward MS , Borg DJ , Gallo LA , Fotheringham AK , Pettit AR , Yap FYT *et al*. (2014) Deletion of bone‐marrow‐derived receptor for AGEs (RAGE) improves renal function in an experimental mouse model of diabetes. Diabetologia 57, 1977–1985. 24957662 10.1007/s00125-014-3291-z

[42] Ramos MV , Auvynet C , Poupel L , Rodero M , Mejias MP , Panek CA , Vanzulli S , Combadiere C and Palermo M (2012) Chemokine receptor CCR1 disruption limits renal damage in a murine model of hemolytic uremic syndrome. Am J Pathol 180, 1040–1048. 22203055 10.1016/j.ajpath.2011.11.011

[43] Schwalm S , Pfeilschifter J and Huwiler A (2014) Targeting the sphingosine kinase/sphingosine 1‐phosphate pathway to treat chronic inflammatory kidney diseases. Basic Clin Pharmacol Toxicol 114, 44–49. 23789924 10.1111/bcpt.12103

[44] Tang H , Yang M , Liu Y , Liu H , Sun L and Song P (2021) The CXCL1‐CXCR2 axis mediates tubular injury in diabetic nephropathy through the regulation of the inflammatory response. Front Physiol 12, 782677. 34975537 10.3389/fphys.2021.782677 PMC8716832

[45] Deng Z , Jing Z , Guo Y , Ma J , Yan H , Shi Z , Deng H , Liang Y , Wang S , Cui Z *et al*. (2021) Expression of immunoglobulin G in human proximal tubular epithelial cells. Mol Med Rep 23, 327. 33760139 10.3892/mmr.2021.11966 PMC7974459

[46] Faron‐Górecka A , Kuśmider M , Kolasa M , Zurawek D , Gruca P , Papp M , Szafran K , Solich J , Pabian P , Romańska I *et al*. (2014) Prolactin and its receptors in the chronic mild stress rat model of depression. Brain Res 1555, 48–59. 24508286 10.1016/j.brainres.2014.01.031

[47] Roy M , David N , Cueva M and Giorgetti M (2007) A study of the involvement of melanin‐concentrating hormone receptor 1 (MCHR1) in murine models of depression. Biol Psychiatry 61, 174–180. 16934771 10.1016/j.biopsych.2006.03.076

[48] Wu W , Sun M , Zhang HP , Chen T , Wu R , Liu C , Yang G , Geng XR , Feng BS , Liu Z *et al*. (2014) Prolactin mediates psychological stress‐induced dysfunction of regulatory T cells to facilitate intestinal inflammation. Gut 63, 1883–1892. 24550371 10.1136/gutjnl-2013-306083 PMC4251191

[49] Leung YT , Maurer K , Song L , Convissar J and Sullivan KE (2020) Prolactin activates IRF1 and leads to altered balance of histone acetylation: implications for systemic lupus erythematosus. Mol Rheumatol 20, 532–543. 10.1080/14397595.2019.1620999 31104557

[50] Kim TK , Kim JE , Park JY , Lee JE , Choi J , Kim H , Lee EH , Kim SW , Lee JK , Kang HS *et al*. (2015) Antidepressant effects of exercise are produced via suppression of hypocretin/orexin and melanin‐concentrating hormone in the basolateral amygdala. Neurobiol Dis 79, 59–69. 25917762 10.1016/j.nbd.2015.04.004

[51] Hamamoto A , Yamato S , Katoh Y , Nakayama K , Yoshimura K , Takeda S , Kobayashi Y and Saito Y (2016) Modulation of primary cilia length by melanin‐concentrating hormone receptor 1. Cell Signal 28, 572–584. 26946173 10.1016/j.cellsig.2016.02.018

[52] Kong X , Ota SM , Suchecki D , Lan A , Peereboom AI , Hut RA and Meerlo P (2022) Chronic social defeat stress shifts peripheral circadian clocks in male mice in a tissue‐specific and time‐of‐day dependent fashion. J Biol Rhythms 37, 164–176. 34994236 10.1177/07487304211065336

[53] Liu L , Pang XL , Shang WJ , Xie HC , Wang JX and Feng GW (2018) Over‐expressed microRNA‐181a reduces glomerular sclerosis and renal tubular epithelial injury in rats with chronic kidney disease via down‐regulation of the TLR/NF‐κB pathway by binding to CRY1. Mol Med 24, 49. 30241461 10.1186/s10020-018-0045-2 PMC6145098

[54] Ibrahim EC , Gorgievski V , Ortiz‐Teba P , Belzeaux R , Turecki G , Sibille E , Charbonnier G and Tzavara ET (2022) Transcriptomic studies of antidepressant action in rodent models of depression: a first meta‐analysis study. Int J Mol Sci 23, 13543. 36362329 10.3390/ijms232113543 PMC9654684

[55] Qin Z , Wang H , Dou Q , Xu L , Xu Z and Jia R (2022) Protective effect of fluoxetine against oxidative stress induced by renal ischemia‐reperfusion injury via the regulation of miR‐450b‐5p/Nrf2 axis. Aging 14, doi: 10.18632/aging.204289 PMC10781502 36126189

[56] Liu D , Wang Z , Liu S , Wang F , Zhao S and Hao A (2011) Anti‐inflammatory effects of fluoxetine in lipopolysaccharide(LPS)‐stimulated microglial cells. Neuropharmacology 61, 592–599. 21575647 10.1016/j.neuropharm.2011.04.033

[57] Jazayeri S , Keshavarz SA , Tehrani‐Doost M , Djalali M , Hosseini M , Amini H , Chamari M and Djazayery A (2010) Effects of eicosapentaenoic acid and fluoxetine on plasma cortisol, serum interleukin‐1beta and interleukin‐6 concentrations in patients with major depressive disorder. Psychiatry Res 178, 112–115. 20466437 10.1016/j.psychres.2009.04.013

[58] Jorgensen H , Kjaer A , Warberg J and Knigge U (2001) Differential effect of serotonin 5‐HT(1A) receptor antagonists on the secretion of corticotropin and prolactin. Neuroendocrinology 73, 322–333. 11399905 10.1159/000054649

[59] Calegare BF , Costa A , Fernandes L , Dias AL , Torterolo P and D'Almeida V (2016) Subchronical treatment with fluoxetine modifies the activity of the MCHergic and hypocretinergic systems. Evidences from peptide CSF concentration and gene expression. Sleep Sci 9, 89–93. 27656272 10.1016/j.slsci.2016.05.010 PMC5022008

[60] Cuesta M , Clesse D , Pévet P and Challet E (2009) New light on the serotonergic paradox in the rat ciecadian system. J Neurochem 110, 231–243. 19457131 10.1111/j.1471-4159.2009.06128.x

[61] Pallier PN , Ferrara M , Romagnolo F , Ferretti MT , Soreq H and Cerase A (2022) Chromosomal and environmental contributions to sex differences in the vulnerability to neurological and neuropsychiatric disorders: implications for therapeutic interventions. Prog Neurobiol 219, 102353. 36100191 10.1016/j.pneurobio.2022.102353

[62] Kauffman K , Dolata J , Figueroa M , Gunzler D , Huml A , Pencak J , Sajatovic M and Sehgal AR (2022) Directly observed weekly fluoxetine for major depressive disorder among hemodialysis patients: a single‐arm feasibility trial. Kidney Med 4, 100413. 35386606 10.1016/j.xkme.2022.100413 PMC8978139

[63] Vieira‐Coelho MA and Martel F (2023) Inhibition of kidney potassium channels by fluoxetine: in vivo and in vitro studies. Fundam Clin Pharmacol 37, 226–234. 36103995 10.1111/fcp.12833

[64] Ghavamabadi RT , Taghipour Z , Hassanipour M , Khademi M and Shariati M (2018) Effect of maternal fluoxetine exposure on lung, heart, and kidney development in rat neonates. Iran J Basic Med Sci 21, 417–421. 29796227 10.22038/IJBMS.2018.27203.6650 PMC5960760

[65] Harris AZ , Atsak P , Bretton ZH , Holt ES , Alam R , Morton MP , Abbas AI , Leonardo ED , Bolkan SS , Hen R *et al*. (2018) A novel method for chronic social defeat stress in female mice. Neuropsychopharmacology 43, 1276–1283. 29090682 10.1038/npp.2017.259 PMC5916350

[66] Yohn CN , Dieterich A , Bazer AS , Maita I , Giedraitis M and Samuels BA (2019) Chronic non‐discriminatory social defeat is an effective chronic stress paradigm for both male and female mice. Neuropsychopharmacology 44, 2220–2229. 31493767 10.1038/s41386-019-0520-7 PMC6898575

